# Oxidative stress-mediated TXNIP loss causes RPE dysfunction

**DOI:** 10.1038/s12276-019-0327-y

**Published:** 2019-10-15

**Authors:** Min Ji Cho, Sung-Jin Yoon, Wooil Kim, Jongjin Park, Jangwook Lee, Jong-Gil Park, Young-Lai Cho, Jeong Hun Kim, Hyejin Jang, Young-Jun Park, Sang-Hyun Lee, Jeong-Ki Min

**Affiliations:** 1 Biotherapeutics Translational Research Center, Korea Research Institute of Bioscience and Biotechnology (KRIBB), 125 Gwahak-ro, Yuseong-gu, Daejeon, 34141 Republic of Korea; 2 Department of Biomolecular Science, KRIBB School of Bioscience, Korea University of Science and Technology (UST), 217 Gajeong-ro, Yuseong-gu, Daejeon, 34141 Republic of Korea; 3 Environmental Disease Research Center, Korea Research Institute of Bioscience and Biotechnology (KRIBB), 125 Gwahak-ro, Yuseong-gu, Daejeon, 34141 Republic of Korea; 4 Metabolic Regulation Research Center, Korea Research Institute of Bioscience and Biotechnology (KRIBB), 125 Gwahak-ro, Yuseong-gu, Daejeon, 34141 Republic of Korea; 5 Fight against Angiogenesis-Related Blindness (FARB) Laboratory, Clinical Research Institute, Seoul National University Hospital, 101 Daehak-ro, jongno-gu, Seoul, 03080 Republic of Korea

**Keywords:** Stress signalling, Macroautophagy

## Abstract

The disruption of the retinal pigment epithelium (RPE), for example, through oxidative damage, is a common factor underlying age-related macular degeneration (AMD). Aberrant autophagy also contributes to AMD pathology, as autophagy maintains RPE homeostasis to ensure blood–retinal barrier (BRB) integrity and protect photoreceptors. Thioredoxin-interacting protein (TXNIP) promotes cellular oxidative stress by inhibiting thioredoxin reducing capacity and is in turn inversely regulated by reactive oxygen species levels; however, its role in oxidative stress-induced RPE cell dysfunction and the mechanistic link between TXNIP and autophagy are largely unknown. Here, we observed that TXNIP expression was rapidly downregulated in RPE cells under oxidative stress and that RPE cell proliferation was decreased. TXNIP knockdown demonstrated that the suppression of proliferation resulted from TXNIP depletion-induced autophagic flux, causing increased p53 activation via nuclear localization, which in turn enhanced AMPK phosphorylation and activation. Moreover, TXNIP downregulation further negatively impacted BRB integrity by disrupting RPE cell tight junctions and enhancing cell motility by phosphorylating, and thereby activating, Src kinase. Finally, we also revealed that TXNIP knockdown upregulated HIF-1α, leading to the enhanced secretion of VEGF from RPE cells and the stimulation of angiogenesis in cocultured human retinal microvascular endothelial cells. This suggests that the exposure of RPE cells to sustained oxidative stress may promote choroidal neovascularization, another AMD pathology. Together, these findings reveal three distinct mechanisms by which TXNIP downregulation disrupts RPE cell function and thereby exacerbates AMD pathogenesis. Accordingly, reinforcing or restoring BRB integrity by targeting TXNIP may serve as an effective therapeutic strategy for preventing or attenuating photoreceptor damage in AMD.

## Macular degeneration: crucial protein identified

A protein found in retinal cells promotes the development of age-related macular degeneration and may provide a therapeutic target. Sight loss through macular degeneration is triggered by disruption to the retinal pigment epithelium (RPE), a layer of cells that carries nutrients to the eye. RPE cells can be disrupted under oxidative stress conditions, but how this influences macular degeneration is unclear. Jeong-Ki Min and Sang-Hyun Lee at the Korea Research Institute of Bioscience and Biotechnology in Daejeon, South Korea, and co-workers found that oxidative stress reduces levels of the thioredoxin-interacting protein (TXNIP) in human RPE cell cultures. This interrupts cellular communication and disturbs the balance between cell proliferation and cell recycling. It also increases the levels of proteins that promote excess blood vessel formation, a key process contributing to macular degeneration.

## Introduction

Age-related macular degeneration (AMD) constitutes a progressive, chronic disease that represents a common irreversible cause of severe loss of vision^1^. Vision loss in AMD occurs through photoreceptor damage in the macula. In particular, cellular dysfunction of the retinal pigment epithelium (RPE), a major component of the blood-retinal barrier (BRB), might lead to the disruption of photoreceptor homeostasis^2,3^. In the later stage of AMD, patients suffer from macular damage, which can occur as a consequence of geographic atrophy or choroidal neovascularization (CNV). In geographic atrophy, the RPE degenerates, leading to a progressive loss of photoreceptors. The wet form of AMD is characterized by the abnormal growth of new blood vessels that break through the BRB and grow into the retina under the macula^4^. RPE damage and a loss of BRB function comprise common features of both dry and wet AMD, in which vascular endothelial growth factor (VEGF) plays a major pathogenic role^5^.

The RPE is particularly metabolically active, highly oxygenated, and vulnerable to oxidative damage because it is exposed to photosensitizers such as antioxidants and lipofuscin. This sensitivity leads to a variety of age-related changes that reduce RPE function and increase cell death^6^. Among the risk factors for AMD, oxidative stress serves as a key component of AMD pathogenesis. Oxidative stress refers to the progressive cell damage caused by reactive oxygen species (ROS), which contribute to protein misfolding and evoke dysfunction during RPE cell senescence^7^. Numerous studies have reported that the cumulative amount of damage increases with age due to impairments in the DNA repair system along with intensified oxidative stress and decreased antioxidant defense. Moreover, the much less effective recovery systems for mitochondrial DNA damage can cause oxidative stress and the accumulation of the resulting aberrant products^8,9^. However, little is known regarding the specific factors that mediate oxidative stress in RPE cells. One potential candidate, autophagy, is especially crucial for the maintenance of RPE homeostasis, as RPE cells are exposed to sustained oxidative stress^10^. In the pathogenesis of AMD, insufficient digestion resulting from impaired autophagy in the RPE leads to the accumulation of damaged organelles, extracellular drusen deposits, and lipofuscin. However, once the RPE is damaged beyond a critical point, such as in the later stage of AMD, functional autophagy might instead cause cell death and exacerbate the disease^11^.

Thioredoxin-interacting protein (TXNIP), an α-arrestin family member, acts as a multifunctional adaptor protein for different signaling pathways^12^. The primary role of TXNIP is the negative regulation of thioredoxin (TRX) function by inhibiting its reducing capacity and promoting cellular oxidative stress^13^. The inhibition of TRX by TXNIP carries lethal consequences for cells and accelerates destructive inflammation^14^. In human aortic endothelial cells with TXNIP knockdown cultured under high-glucose conditions to promote oxidative stress, a decrease in the amount of ROS generated was observed compared with that observed in control cells. This suggests that high levels of TXNIP inhibit the redox activity of cytoplasmic TRX1 associated with an increase in ROS levels^15^. Conversely, ROS appear to negatively regulate TXNIP expression in vascular smooth muscle cells, as the pretreatment of these cells with antioxidative agents inhibits the TXNIP downregulation that occurs following H_2_O_2_ stimulation^16^. These results demonstrate the importance of ROS for the expression of TXNIP^16^.

p53 is a well-known tumor suppressor with the ability to cause cell senescence and apoptosis^17^. Although p53 can indirectly influence cell growth and proliferation by activating cell cycle inhibitors or the transcriptional activation of proapoptotic proteins^18,19^, p53 also regulates autophagy^20–22^. For example, autophagy can be induced in a p53-dependent manner in response to genotoxins. p53-induced autophagy occurs through the activation of AMP-activated kinase (AMPK), which results in the rapid, acute inhibition of the mammalian target of rapamycin complex 1 (mTORC1) through the activation of tuberous sclerosis (TSC) 1 and 2 kinases. In addition, p53 activation can also contribute to the long-term inhibition of mTOR by inducing the upregulation of phosphatase and tensin homologue and TSC2 at the transcriptional level^23^. Another mechanism of p53-induced autophagy involves the transcriptional activation of damage-regulated autophagy modulator^20^. Autophagy induced by p53 may facilitate p53 cell cycle arrest activity and synergize with accelerated cell death in response to p53 activation. However, the role of TXNIP in oxidative stress-induced RPE cell dysfunction and the mechanistic link between TXNIP and autophagy remain to be clarified.

In this study, we observed that TXNIP expression was rapidly downregulated in RPE cells under oxidative stress. Notably, we showed that the knockdown of TXNIP induces autophagic flux through increased p53 activation. Furthermore, we demonstrated that TXNIP is involved in neovascularization through the regulation of hypoxia-inducible factor-1α (HIF-1α) activation.

## Materials and methods

### Cell culture and transfection

The human retinal pigment epithelium cell line ARPE-19 was purchased from ATCC. The ARPE-19 cells were cultured in Dulbecco’s modified Eagle’s medium/nutrient mixture F-12 (DMEM/F-12, Gibco) supplemented with 10% fetal bovine serum (FBS) and 1% penicillin/streptomycin in a humidified incubator at 37 °C. shTXNIP (NM_006472.3-1666s21c1) and non-target shRNA control (SHC002) cloned in a PLKO.1 lentiviral vector were purchased from Sigma (St. Louis, USA). The overexpression of TXNIP in ARPE-19 cells was achieved by lentivirus-mediated transduction of full-length human *TXNIP* subcloned into a pLKO.1 and a pLVX-EF1α-IRES-Puro lentiviral vector (Clontech, USA). To generate stable transfectants, the lentiviral vector was cotransfected into Lenti-X-293T (Clontech) cells with virus packaging mix (Sigma) using Lipofectamine 2000 reagent (Invitrogen) according to the manufacturer’s instructions. The virus, along with 5 µg/ml polybrene (Santa Cruz Biotechnology), was added to ARPE-19 cells. After 20 h, the medium was removed and replaced with fresh media containing 3 μg/ml puromycin (Santa Cruz Biotechnology). Puromycin-resistant clones were selected by culturing for 2 weeks in the presence of puromycin. TXNIP expression levels were analyzed by western blotting. For rescue experiments, RNAi-resistant human eGFP-TXNIP was transfected into TXNIP KD cells. The cells were transfected with GFP-LC3 and mRFP-GFP-LC3 with Lipofectamine 2000 (Invitrogen) according to the manufacturer’s instructions and cultured for 12 h. All experiments were performed 32 h after transfection. siRNA against human *LC3* and *p53* and nonspecific control siRNA were purchased from Santa Cruz Biotechnology. For siRNA experiments, 1 × 10^6^ cells were transfected with 100 pmol of control siRNA, siLC3 and sip53 using the Neon transfection system (Invitrogen) (conditions: 1600 V, 10 ms, 2 pulses) and then cultured for 48 h.

### DNA constructs

To overexpress TXNIP in ARPE-19 cells, TXNIP was generated by PCR amplification and inserted into a pLVX-EF1α-IRES-Puro lentiviral vector or a pEGFP-C1 vector. TXNIP DNA was amplified using the following primer sets: 5’-GCG AAT TCG ATG GTG ATG TTC AAG AAG ATC-3’ and 5’-CCG TCT GAG TCA CTG CAC ATT GTT GTT GAG-3’ (the amplified fragments were ligated into the EcoRI/XbaI sites of the pLVX-EF1α-IRES-Puro lentiviral vector); 5’-GCG AAT TCG ATG GTG ATG TTC AAG AAG ATC-3’ and 5’-CCG GGT ACC TCA CTG CAC ATT GTT GTT GAG-3’ (the amplified fragments were ligated into the EcoRI/KpnII sites of the pEGFP-C1 vector).

### Cell viability assay

The cytotoxicity of H_2_O_2_ was assessed by an MTT (M5655, Sigma-Aldrich, USA) assay. Cells (1 × 10^4^ cells/well) were seeded into 96-well plates. After overnight incubation, the culture medium was removed, the cells were rinsed with phosphate buffered saline (PBS), and the cells were treated with the indicated concentration of H_2_O_2_ in culture medium containing 1% FBS. After 24 h of H_2_O_2_ treatment, 0.5 mg/ml MTT was added to each well and incubated for 4 h to allow mitochondrial dehydrogenase to convert MTT to insoluble formazan crystals. At the end of treatment, MTT was added to the culture medium for 4 h. The medium was then aspirated, and the formazan was solubilized by the addition of 100 μl of DMSO. The absorbance at 570 nm was measured using an enzyme-linked immunosorbent assay (ELISA) microplate reader. Each experiment was performed in triplicate and repeated three times to assess the reproducibility of the results.

### Cell proliferation assay

The proliferation of wild-type (WT), shCtrl, and shTXNIP ARPE-19 cells was determined using a Wst-1 assay. Cells (1 × 10^4^ cells/well) were seeded into 96-well plates. After overnight incubation, the culture medium was removed, and the cells were rinsed with phosphate-buffered saline (PBS). The cells were treated with or without H_2_O_2_ in culture medium containing 5% FBS. After a certain period of time (24, 48, or 72 h), 10 μl of Wst-1 reagent (ab155902, Abcam, USA) was added to each well. After incubation with Wst-1 reagent for 2 h, the medium was collected, and the absorbance of the treated and untreated samples was measured using an ELISA microplate reader at 440 nm. Each experiment was performed in triplicate and repeated three times to assess the reproducibility of the results.

### Cell cycle analysis

The shCtrl and shTXNIP ARPE-19 cells, were cultured in normal growth medium for 48 h. After 48 h, the cells (4 × 10^5^ cells/well) were detached and resuspended in PBS. The cells were stained with 5 μM Draq5^TM^ (#424101, BioLegend, USA) for 15 min at room temperature. After incubation with Draq5^TM^, the cells were spun down and washed with PBS. After the cells were resuspended, DNA content was directly analyzed by FACS.

### Cell random motility

The random motility of the cells was determined using specialized bottom-glass confocal dishes (Ibidi, Munich, Germany). Cell suspensions (100 μl) at a density of 1 × 10^4^ cells were seeded in confocal dishes for live-cell motility analyses. After the cells were incubated for 6 h, the dishes were transferred to a live-cell incubating chamber (Live Cell Instrument, Seoul, South Korea) at 37 °C under 5% CO_2_ on the stage of an inverted fluorescence microscope (Olympus, IX81-ZDC) with a U*PLSAPO* 20X objective lens. Random cell motility was monitored over a 4-h period by capturing images every 10 min; data analysis was performed using MetaMorph version 7.1 (Universal Imaging, Media, PA).

### Western blotting

Cells were washed in PBS and solubilized in RIPA buffer (50 mM Tris-HCl, 150 mM NaCl, 1% NP-40, 0.1% sodium dodecyl sulfate [SDS], and 0.5% sodium deoxycholate) supplemented with proteinase and phosphatase inhibitor cocktails (GenDEPOT). The cell lysates were centrifuged for 10 min at 13,000 rpm at 4 °C to remove cellular debris. The protein contents of the cells were determined, and the cellular lysates were separated by SDS-polyacrylamide gel electrophoresis and electrotransferred to polyvinylidene difluoride membranes. After being blocked in TBST with 5% nonfat milk, the membranes were incubated overnight with primary antibodies, including anti-TXNIP (K0205, 1:1000, MBL life, USA), anti-Trx (ab26320, 1:2000, Abcam), anti-β-actin (AbC-2004, 1:5000, AbClon, South Korea), anti-LC3B (L7543, 1:5000, Sigma), anti-MDM2 (sc-13161, 1:1000, Santa Cruz, USA), anti-HIF-1a (NB100-105, 1: 1000, Novus Biologicals, USA), anti-p53 (sc-126, 1:2000, Santa Cruz), anti-cyclin A (sc-751, 1:1000, Santa Cruz), anti-cyclin D1 (sc-8396, 1:1000, Santa Cruz), anti-phospho-AMPKα (#2531, 1:1000, Cell Signaling, USA), anti-phospho-mTOR (#5536, 1:1000, Cell Signaling), anti-phospho-p53 (#9286, 1:1000, Cell Signaling), anti-phospho-Src (#2101, 1:1000, Cell Signaling), and anti-phospho-FAK (#8556, 1:1000, Cell Signaling), at 4 °C, followed by incubation with a horseradish peroxidase-conjugated secondary antibody (1:1000 dilution) for 1 h. Immunoreactive bands were visualized using an Enhanced Chemiluminescence Kit (Amersham).

### Coimmunoprecipitation (Co-IP) experiments

Cells were lysed in RIPA buffer supplemented with proteinase and phosphatase inhibitor cocktails. The cell lysates were centrifuged for 10 min at 13,000 rpm at 4 °C to remove cellular debris. To preclear the cell lysates, 50 μl of normal serum was added to 500 μg of each cell lysate and mixed with 10 μl of a protein A-conjugated agarose bead slurry. The cell lysates were incubated at 4 °C under rotary agitation for 1 h. After preclearing, the cell lysates were centrifuged at 4000 rpm at 4 °C for 10 min, and the supernatant was kept for the IP experiments. The supernatants were incubated for 4 h with 2.5 μg of an anti-MDM2 or anti-TXNIP antibody at 4 °C under rotary agitation. Next, 80 µl of protein A-conjugated agarose beads was added, and the resulting mixtures were incubated for 8 h at 4 °C under rotary agitation. The immunoprecipitated samples were washed three times with RIPA buffer, eluted with 2× loading buffer at 95 °C for 10 min, and analyzed by western blotting.

### Immunofluorescence assays

To visualize tight junctions, focal adhesions and F-actin formation, cells were attached to glass coverslips coated with 10 μg/ml fibronectin. The cells were washed once in PBS, fixed for 10 min in 3.7% formaldehyde, permeabilized for 20 min at room temperature with 0.2% Triton X-100, washed in PBS, and blocked for 30 min at room temperature in 1% bovine serum albumin in PBS. After incubation for 1 h with a ZO-1 (#13663, 1:1000, Cell Signaling) or paxillin (PM1071, 1:500, ECM Biosciences, USA) antibody, the cells were washed in PBS and incubated for 1 h with FITC-conjugated anti-mouse antibodies and TRITC-labeled phalloidin (P1951, 1:2000, Sigma-Aldrich) in PBS. the cells were washed with PBS and stained with DAPI to visualize the nuclei, and coverslips were mounted on the slides. Images were captured with an Olympus DP30BW digital camera and processed using MetaMorph version 7.1 (Universal Imaging, Media, PA).

### Enzyme-linked immunosorbent assay (ELISA) for VEGF

The VEGF concentration was assayed in the culture medium using a Human VEGF ELISA Kit (Abcam, UK) according to the manufacturer’s instructions. Cell culture medium was collected and supplemented with 2% fetal bovine serum before being stored at −20 °C to maintain the stability of VEGF. All reagents and standards were freshly prepared and added during the assay as instructed by the manufacturer. The concentration of VEGF was measured by the color intensity of the solution using a microplate reader (Spectra Max i3X, Molecular Devices, USA) at 450 nm and 570 nm. The readings at 570 nm were subtracted from the readings at 450 nm to allow the correction of optical imperfections. The VEGF concentration was determined by comparing the corresponding readings with those of the standard curve using known concentrations of VEGF. Each experiment was performed in triplicate and repeated three times to assess the reproducibility of the results.

### Tubule network formation assay

To examine the paracrine effect of VEGF on RPE cells, the transwell assay system was used. In detail, 1.2 × 10^5^ HRMECs were seeded in M199 medium supplemented with 1% FBS in the lower chamber of the transwell compartment coated with growth factor-reduced Matrigel (Corning, NY, USA) in 24-well plates. Then, 1 × 10^6^ RPE cells were seeded in the upper chamber of the transwell compartment (0.4 μm pore size, Corning, NY, USA) coated with 0.1 mg/ml bovine fibronectin. After 18 h, tube networks were observed under bright field microscopy, and the relative tube area was analyzed using ImageJ software.

### Quantification and statistical analysis

Statistical analysis was carried out using Windows Microsoft Excel 2013. Statistical differences between two experimental groups were calculated using the unpaired two-tailed Student’s *t*-test. A significance level of *p* < 0.05 was used throughout the study.

## Results

### The loss of TXNIP constitutes a major cause of the oxidative stress-induced suppression of RPE cell proliferation

**Fig. 1:**
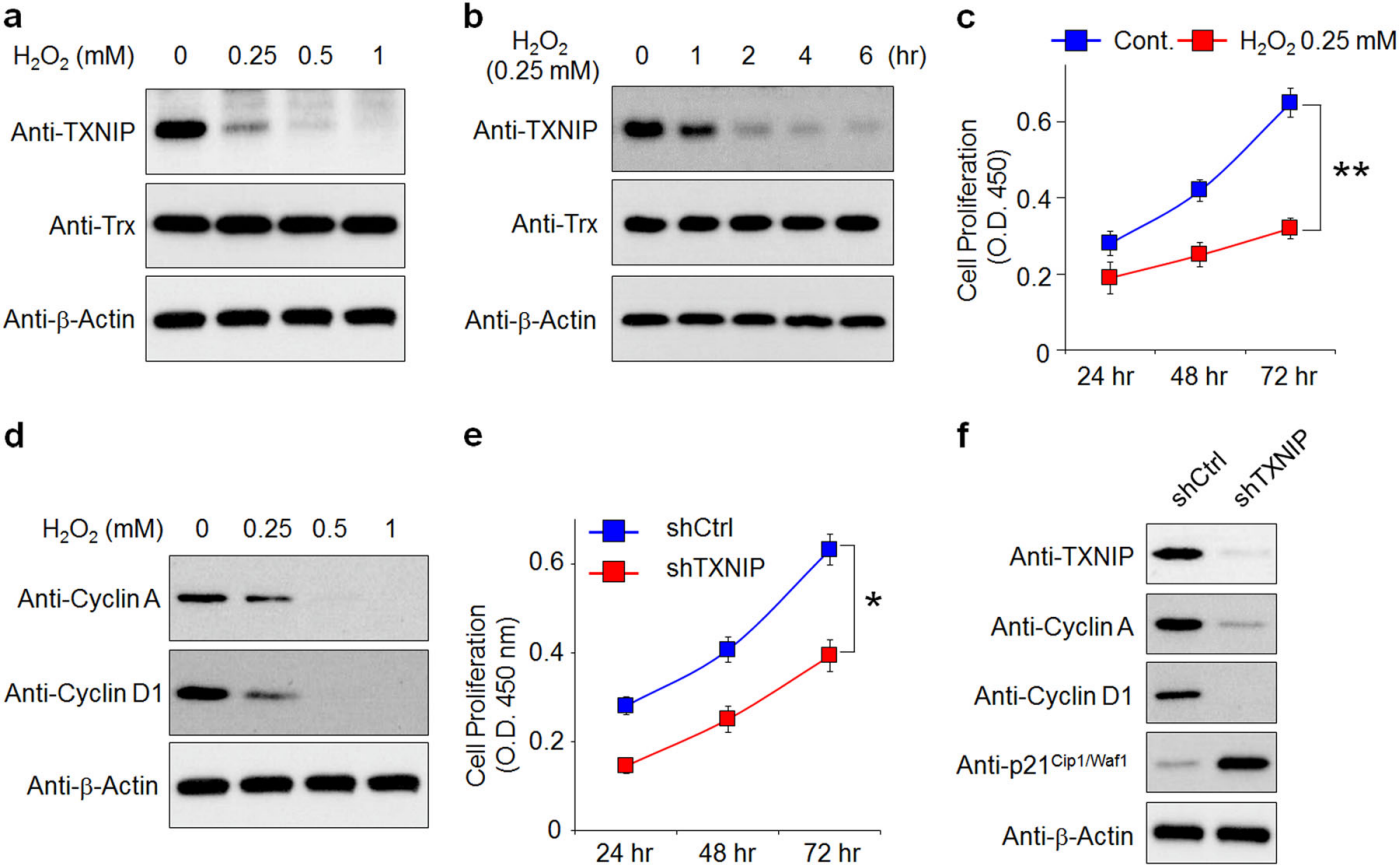
Oxidative stress significantly reduces TXNIP expression and the suppression of RPE cell proliferation. **a** ARPE-19 cells were treated with the indicated concentration of H_2_O_2_ for 4 h. The cells were lysed and subjected to western blotting for the indicated antibodies. **b** ARPE-19 cells were stimulated with 0.25 mM H_2_O_2_ for the indicated times. **c** MTT assay of the viability of ARPE-19 cells after treatment with H_2_O_2_ compared with that of ARPE-19 cells not treated with H_2_O_2_. The results shown are representative of three independent experiments. **d** ARPE-19 cells were treated with the indicated concentration of H_2_O_2_ for 4 h. The cells were lysed and subjected to western blotting for the indicated antibodies. **e** ARPE-19 cells were treated with 0.25 mM H_2_O_2_ for the indicated time periods. Cell proliferation was assessed by the Wst-1 assay. The results shown are representative of three independent experiments. **f** Immunoblot analysis of cell cycle-related markers in shTXNIP cells. β-Actin was used as an internal loading control.*p < 0.05; **p < 0.01. The error bars indicate the SEM

To elucidate the role of TXNIP in RPE cells under chronic oxidative stress, the cytotoxic effect of H_2_O_2_ was first determined. Human ARPE-19 cells were treated with various concentrations of H_2_O_2_ for 48 h. As shown in Supplementary Fig. 1a, no apoptotic (or cytotoxic) effect was observed with a concentration of H_2_O_2_ up to 0.25 mM. We next addressed whether TXNIP expression in RPE cells is regulated under oxidative stress. Treatment with H_2_O_2_ (0.25 mM), a noncytotoxic concentration, rapidly and markedly abrogated the level of TXNIP (Fig. 1a, b) and resulted in a significant decrease in the proliferation in ARPE-19 cells (Fig. 1c). Consistent with decreased proliferation, the expression of cell cycle regulators such as cyclin A and cyclin D1 was reduced in H_2_O_2_-treated ARPE-19 cells (Fig. 1d). To determine whether the loss of TXNIP by H_2_O_2_ is involved in the reduction in RPE cell proliferation, we generated TXNIP knockdown RPE cell lines by the lentivirus-mediated transduction of a TXNIP-specific shRNA (shTXNIP) (Supplementary Fig. 1b). We found that the knockdown of TXNIP significantly decreased the proliferation of ARPE-19 cells (Fig. 1e). Consistent with these results, a marked reduction in cyclin A and cyclin D1 was also observed in TXNIP-depleted ARPE-19 cells, whereas the expression of a cyclin-dependent kinase inhibitor 1 (p21^Cip1/Waf1^) was increased (Fig. 1f). These results indicated that the loss of TXNIP by oxidative stress reduces RPE cell proliferation.

### Inhibition of autophagy attenuates the TXNIP loss-induced suppression of RPE cell proliferation

**Fig. 2:**
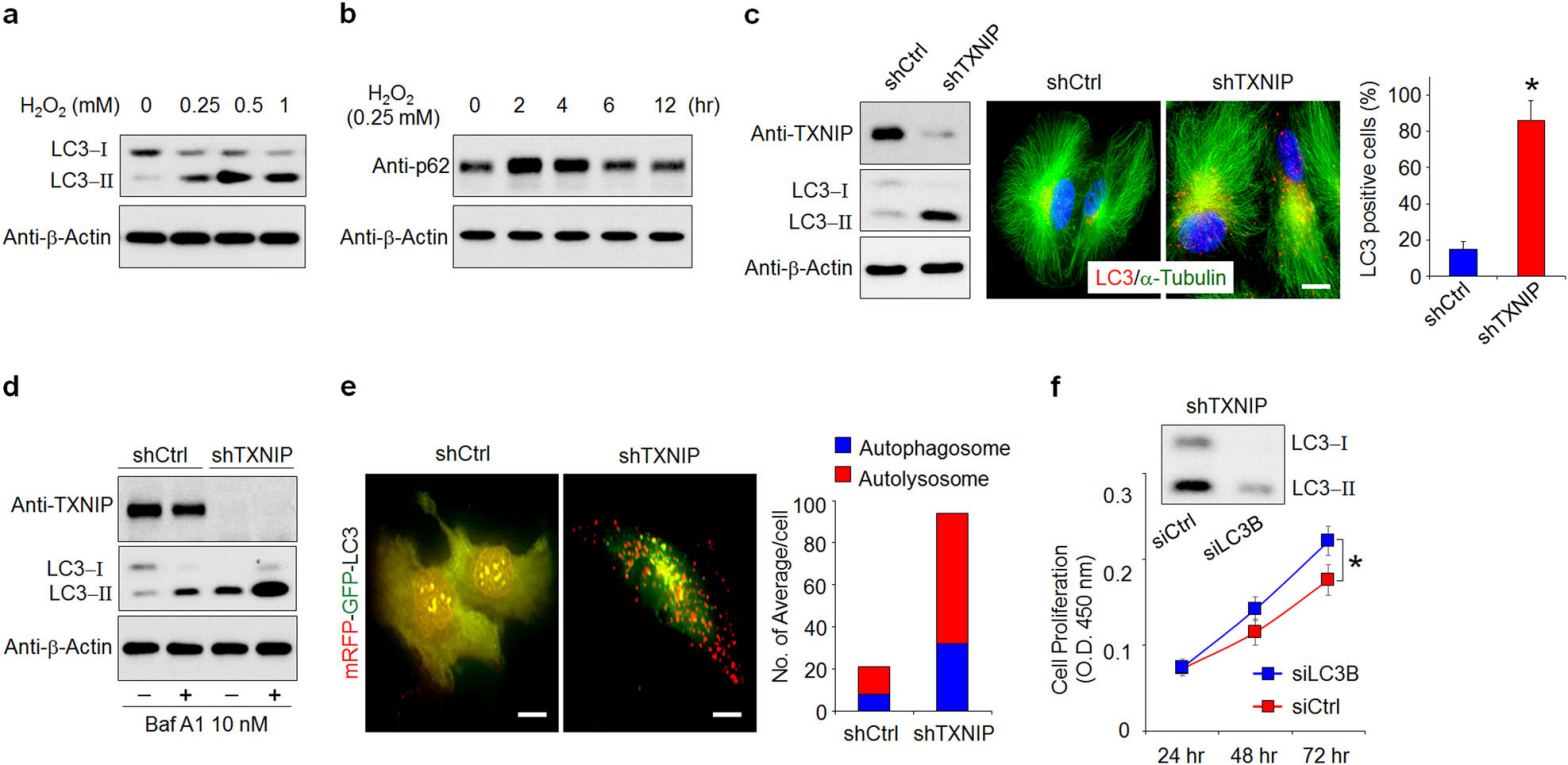
Inhibition of autophagy attenuates the TXNIP loss-induced suppression of RPE cell proliferation. **a** Immunoblot analysis of microtubule-associated protein 1 light chain 3 (LC3)-I to LC3-II conversion in ARPE-19 cells treated with the indicated concentration of H_2_O_2_ for 4 h. The cells were lysed and subjected to western blotting for the indicated antibodies. **b** ARPE-19 cells were stimulated with 0.25 mM H_2_O_2_ for the indicated times. **c** TXNIP-depleted cells displayed notably increased LC3-II accumulation (left panel). RPE cells were plated on coverslips coated with 10 μg/ml fibronectin. Immunostaining was performed on fixed cells with anti-α-tubulin and anti-LC3 antibodies (right panel). LC3-positive puncta (%, the number of LC3 positive cells/the number of total cells) in the images were counted. *p < 0.01 versus shCtrl. **d** The effect of a lysosomal inhibitor, Bafilomycin A1 (Baf A1), on autophagy induction by TXNIP loss in shTXNIP cells. RPE cells were treated with 10 nM Baf A1 for 30 min. The cell lysates were collected and analyzed by western blotting using an anti-LC3 antibody. **e** Representative images of mRFP-GFP-LC3 puncta. The colocalization of GFP and red fluorescent protein (RFP), as indicated by yellow dots in the overlapped GFP and RFP images, was visible in autophagosomes, whereas only RFP fluorescence, indicated by red puncta, was observed in autolysosomes. Autophagosome-positive and autolysosome-positive mRFP-GFP-LC3-transfected cells were counted. **f** The depletion of LC3 attenuated the TXNIP loss-mediated suppression of RPE cell proliferation. shTXNIP cells were transfected with siCtrl or siLC3B and cultured for the indicated time periods. Cell proliferation was performed by the Wst-1 assay. *p < 0.01. The error bars indicate the SEM. Scale bars, 50 μm

Increasing evidence has shown that autophagy and proliferation can act cooperatively, antagonistically, or synergistically to regulate cellular homeostasis^10^. To explore whether the TXNIP loss-induced suppression of proliferation in RPE cells results from an interplay between autophagy and proliferation, we first evaluated the effects of oxidative stress on the induction of autophagy in ARPE-19 cells. The conversion of microtubule-associated protein 1 A/1B-light chain 3 (LC3)-I to LC3-II was markedly increased in a dose-dependent manner in ARPE-19 cells treated with H_2_O_2_ (Fig. 2a). Moreover, the total cellular expression levels of p62 were time-dependently decreased in 0.25 mM H_2_O_2_-treated RPE cells, which is indicative of an increase in autophagic activity, as p62 is selectively incorporated into the autophagosome through direct binding to LC3 (Fig. 2b). Autophagy induction was further supported by the increased number of cytoplasmic punctae expressing green fluorescent protein (GFP)-LC3 fusion proteins in RPE cells (Supplementary Fig. 2a). In contrast, a ubiquitous, diffuse pattern of cytosolic green fluorescence was observed in untreated RPE cells.

Next, to determine whether the loss of TXNIP is involved in oxidative stress-induced autophagy, we examined the conversion and formation of LC3 in shTXNIP cells. The conversion of LC3 and the number of cytoplasmic punctae were notably increased in shTXNIP cells compared with shCtrl cells (Fig. 2c). In contrast, the overexpression of TXNIP in RPE cells markedly blocked H_2_O_2_-induced LC3 conversion (Supplementary Fig. 2b, c). Although the accumulation of LC3-II or an increased number of cytoplasmic LC3 punctae indicates the induction of autophagy, this phenomenon may result from the interruption of autophagolysomal maturation or the completion of autophagy^24^. Thus, we performed turnover assays for LC3 to determine whether the overall autophagic flux was induced. As shown in Fig. 2d, TXNIP-depleted ARPE-19 cells exhibited an increase in LC3-II accumulation in the presence of bafilomycin A1, a lysosomal inhibitor, indicating that an increased amount of LC3 in autophagosomes was delivered to lysosomes for degradation. In addition, based on the difference in acidic stability between GFP and red fluorescence protein, TXNIP-depleted ARPE-19 cells displayed more yellow and red puncta compared with that in shCtrl cells, clearly indicating that autophagic flux was increased in TXNIP-depleted ARPE-19 cells (Fig. 2e). To determine the role of TXNIP loss-induced autophagy, TXNIP-depleted ARPE-19 cells were transfected with LC3-specific small interfering RNA (siRNA) or control siRNA (siCtrl), and their respective cell proliferation was compared. LC3-specific siRNA treatment significantly decreased the TXNIP loss-induced suppression of proliferation in TXNIP-depleted ARPE-19 cells (Fig. 2f). These results demonstrate that TXNIP loss-induced autophagy is involved in the suppression of RPE cell proliferation under oxidative stress.

### TXNIP loss-mediated p53 activation regulates autophagy

**Fig. 3:**
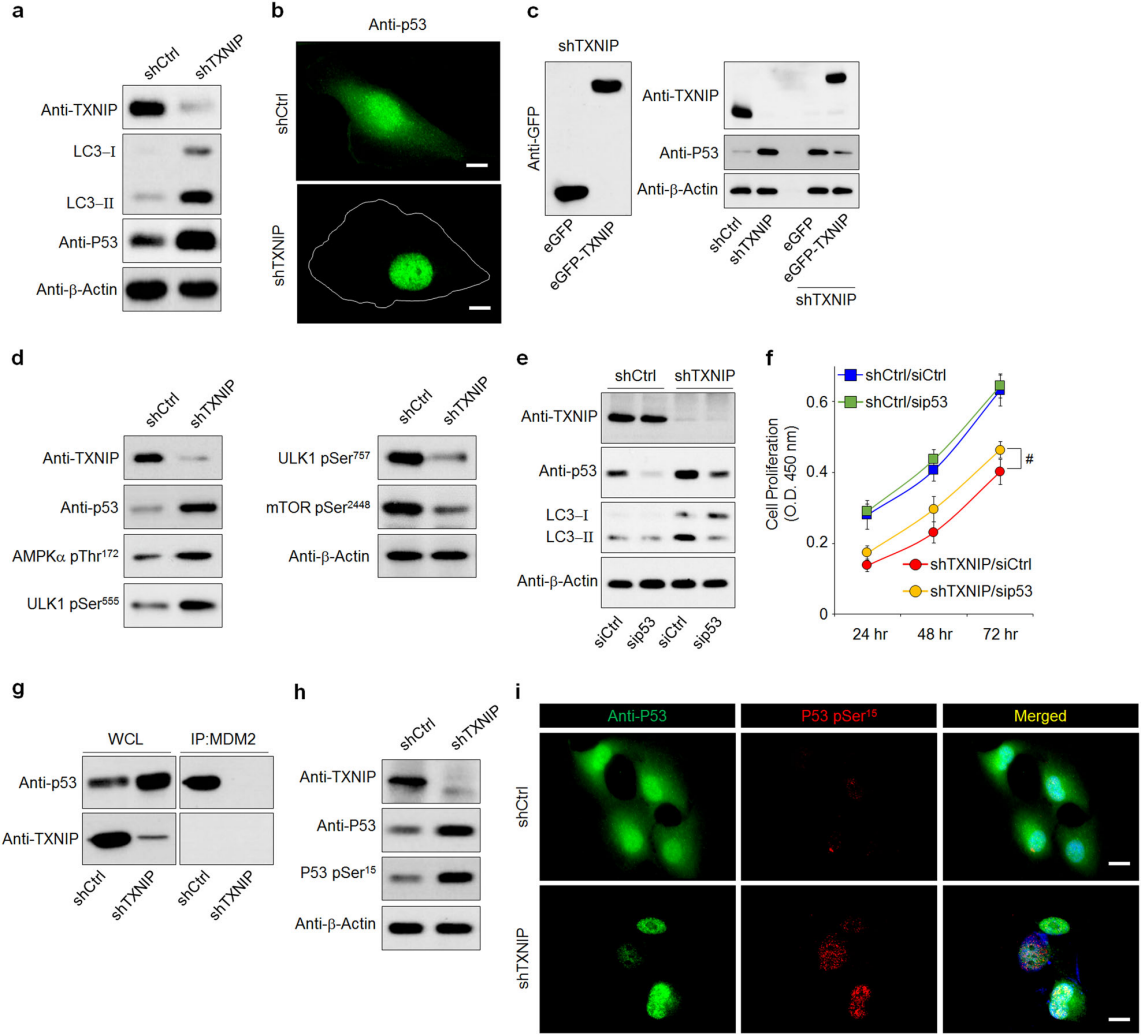
TXNIP loss-mediated p53 activation regulates autophagy. **a** LC3 and p53 expression levels were significantly increased in shTXNIP cells. Cells were lysed and subjected to western blotting using the indicated antibodies. **b** RPE cells were plated on coverslips coated with 10 μg/ml fibronectin. Immunostaining was performed on fixed cells with an anti-p53 antibody. Scale bars, 50 μm. **c** shTXNIP cells were transfected with eGFP or eGFP-TXNIP constructs. The cells were lysed and blotted with the indicated antibodies. **d** The phosphorylation of AMPK at Thr^172^ and ULK1 at Ser^555^ was increased in shTXNIP cells, but the phosphorylation of mTOR at Ser^2448^ and ULK1 at Ser^757^ was decreased. **e** RPE cells were transfected with 10 μM p53 siRNA for 48 h. Cells were lysed and blotted with the indicated antibodies. **f** RPE cells were transfected with siCtrl or siLC3B and cultured for the indicated time periods. Cell proliferation was analyzed by the Wst-1 assay. **g** Cell lysates were immunoprecipitated with an anti-MDM2 antibody, blotted, and probed with anti-p53 and anti-TXNIP antibodies. **h**, **i** The activation of p53 at Ser^15^ was notably increased in shTXNIP cells. Cells were lysed and subjected to western blotting using the indicated antibodies (**h**). Immunostaining was performed on fixed cells with anti-p53 and phospho-p53 Ser^15^ antibodies. Scale bars, 30 μm (**i**). ^#^*p* < 0.01. The error bars indicate the SEM

Next, we scrutinized the signaling pathways that are critically involved in TXNIP loss-induced autophagy in RPE cells. Among the various signaling regulators, p53 is considered a crucial factor in autophagy regulation^20,23^. To determine whether p53 is involved in TXNIP loss-induced autophagy, we examined the level of p53 in TXNIP-depleted ARPE-19 cells. As shown in Fig. 3a, a marked increase in p53 levels was detected in TXNIP-depleted ARPE-19 cells compared with shCtrl cells. Notably, p53 was specifically observed in the nucleus of TXNIP-depleted ARPE-19 cells (Fig. 3b). Similarly, these phenomena were also observed in H_2_O_2_-treated RPE cells (Supplementary Fig. 3a). To determine whether the increase in p53 is directly regulated by TXNIP, TXNIP-depleted ARPE-19 cells were transfected with eGFP-TXNIP. The reexpression of TXNIP in TXNIP-depleted ARPE-19 cells decreased the TXNIP loss-enhanced p53 level (Fig. 3c), suggesting that TXNIP regulates p53 stability or degradation in RPE cells. Considering that decreased cytoplasmic p53 triggers AMPK activation, thereby inhibiting mTOR/Unc-51-like autophagy activating kinase (ULK1) signaling and inducing autophagy^25^, we next demonstrated that the phosphorylation of AMPK at Thr^172^ and ULK1 at Ser^555^ was increased in TXNIP-depleted ARPE-19 cells, whereas that of mTOR at Ser^2448^ and ULK1 at Ser^757^ was markedly decreased (Fig. 3d). To further clarify the involvement of nuclear p53 in TXNIP loss-induced autophagy, TXNIP-depleted ARPE-19 cells were transfected with p53-specific siRNA. p53 knockdown reduced LC3-II accumulation and resulted in a significant increase in the proliferation of TXNIP-depleted ARPE-19 cells (Fig. 3e, f), indicating that the TXNIP loss-mediated accumulation of nuclear p53 induced autophagy and led to the suppression of RPE cell proliferation.

It has been shown that the binding of p53 to MDM2 promotes p53 proteasomal degradation, thus providing a negative autoregulatory mechanism of p53 stability and activity^26^. Therefore, we examined whether TXNIP is involved in the interaction between MDM2 and p53. Coimmunoprecipitation experiments revealed that MDM2 strongly interacts with p53 but not with TXNIP in RPE cells. However, TXNIP strongly binds p53 (Supplementary Fig. 3b). Notably, the interaction between MDM2 and p53 was markedly suppressed in TXNIP-depleted ARPE-19 cells (Fig. 3g). Moreover, as the phosphorylation of p53 constitutes an important event in the control of p53 stability by blocking its binding with MDM2^27^, we further demonstrated that TXNIP loss resulted in the increased phosphorylation of p53 at Ser^15^ in RPE cells (Fig. 3h, i). Collectively, these results suggested that TXNIP might regulate the interaction between p53 and MDM2 by directly binding to p53 and controlling p53 phosphorylation.

### TXNIP is involved in BRB integrity through the regulation of Src kinase activation

**Fig. 4:**
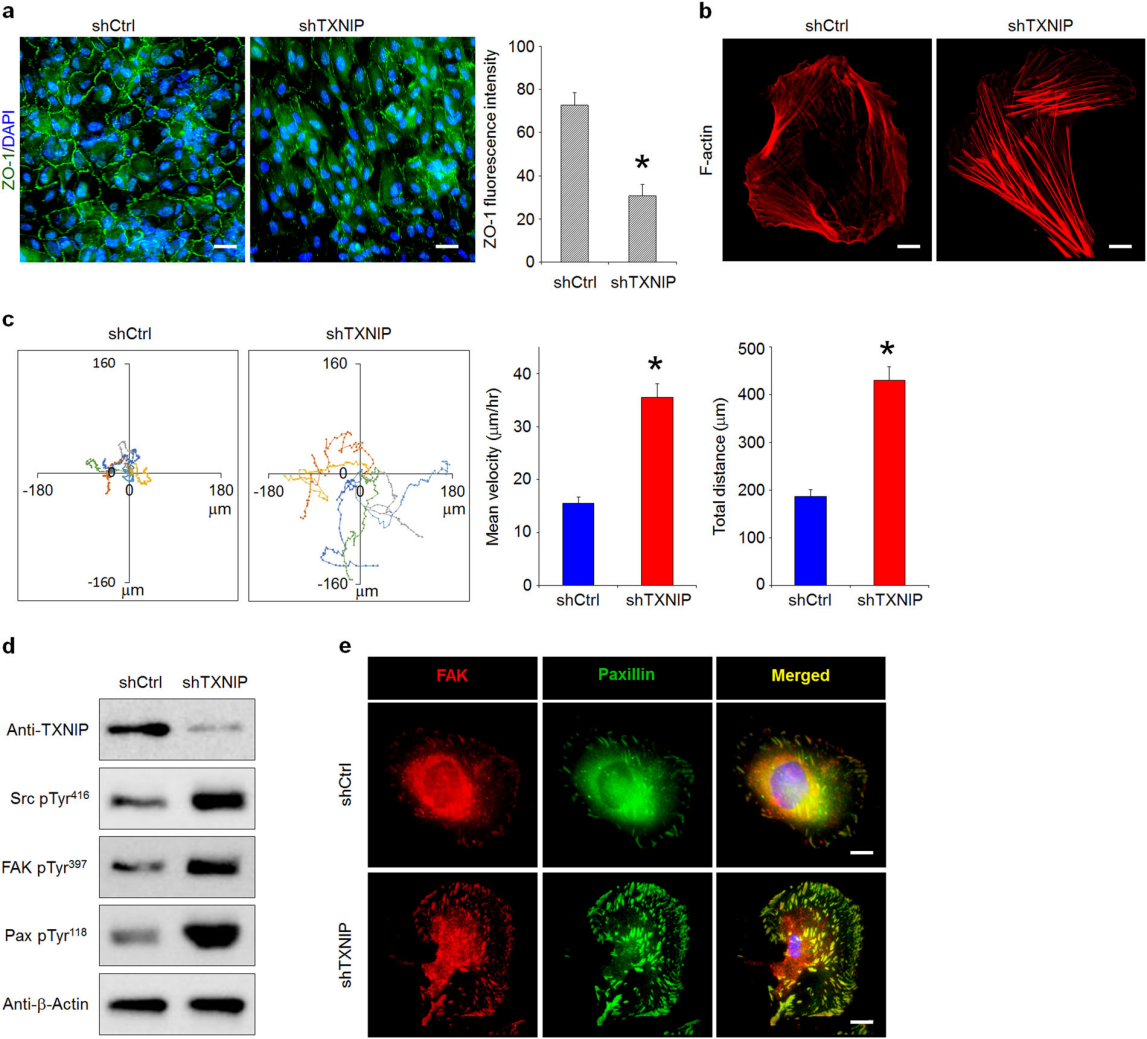
TXNIP is involved in BRB integrity through the regulation of Src kinase activation. **a**, **b** RPE cells were plated on coverslips coated with 10 μg/ml fibronectin. Immunostaining was performed on fixed cells with an anti-ZO1 antibody (**a**) and TRITC-labeled phalloidin (**b**). The fluorescence intensity of ZO-1 along the cell membrane was quantified from the images and is shown in the graphs. **c** Rose plots tracking the movement of six single shCtrl and shTXNIP cells. Each color represents the track of an individual cell. The quantification of velocity and distance from five independent experiments are shown. **d** RPE cell lysates were collected and analyzed by western blotting using the indicated antibodies. **e** Immunostaining was performed on fixed cells with mouse an anti-paxillin antibody and a rabbit anti-FAK antibody. *p < 0.01 versus shCtrl. The error bars indicate the SEM. Scale bars, 30 μm (**a**); and 50 μm (**b** and **e**)

A previous study reported that oxidative stress induces BRB permeability dysfunction through the disruption of tight junctions accompanied by an increase in actin stress fibers in RPE cells^28^. To investigate the role of TXNIP in RPE cell junctional integrity and motility, we performed immunofluorescence analysis. This clearly revealed that zona occludens-1 (ZO-1) was expressed at the cell–cell borders in shCtrl cells, whereas discontinuous or disrupted ZO-1 was observed in TXNIP-depleted ARPE-19 cells (Fig. 4a). Moreover, actin stress fibers were clearly increased in TXNIP-depleted ARPE-19 cells (Fig. 4b), indicating that the disruption of tight junctions resulted from stress fiber-induced tension acting on cell-cell junctions. Next, we examined whether the disruption of tight junctions by TXNIP loss has an effect on cell motility. Single cell random motility analysis revealed enhanced cell motility in TXNIP-depleted ARPE-19 cells (Fig. 4c).

To elucidate the mechanism by which TXNIP regulates RPE cell motility, we examined the activation of focal adhesion kinase (FAK) and Src, as Src kinase activity plays an important role in cell motility and RPE cell integrity^29,30^. TXNIP-depleted cells displayed significantly increased phosphorylation of Src, FAK, and paxillin (Fig. 4d). Moreover, cell locomotion is a dynamic process consisting of repeated cycles of the protrusion of the leading edge, the formation of new matrix adhesions, and the retraction of the trailing edge^31^. In particular, previous reports haves indicated that focal complexes (FXs) are involved in protrusion formation at the leading edge, whereas focal adhesions (FAs) are involved in the contractile actomyosin system to pull the cell body and restrain the migration process^32^. To determine the effect of TXNIP on FX and FA formation, we performed immunostaining for FAK and paxillin. This revealed a significant increase in FX, along with a notable increase in the size of FAs, in the cortex of membranes in TXNIP-depleted ARPE-19 cells (Fig. 4e). These results clearly indicate that the formation of larger, more stable FAs with tethered actin stress fibers, which facilitate cell spreading and are strengthened by Src kinase activation, lead to the disruption of BRB junctional integrity.

### Loss of TXNIP increases HIF-1α and VEGF expression

**Fig. 5:**
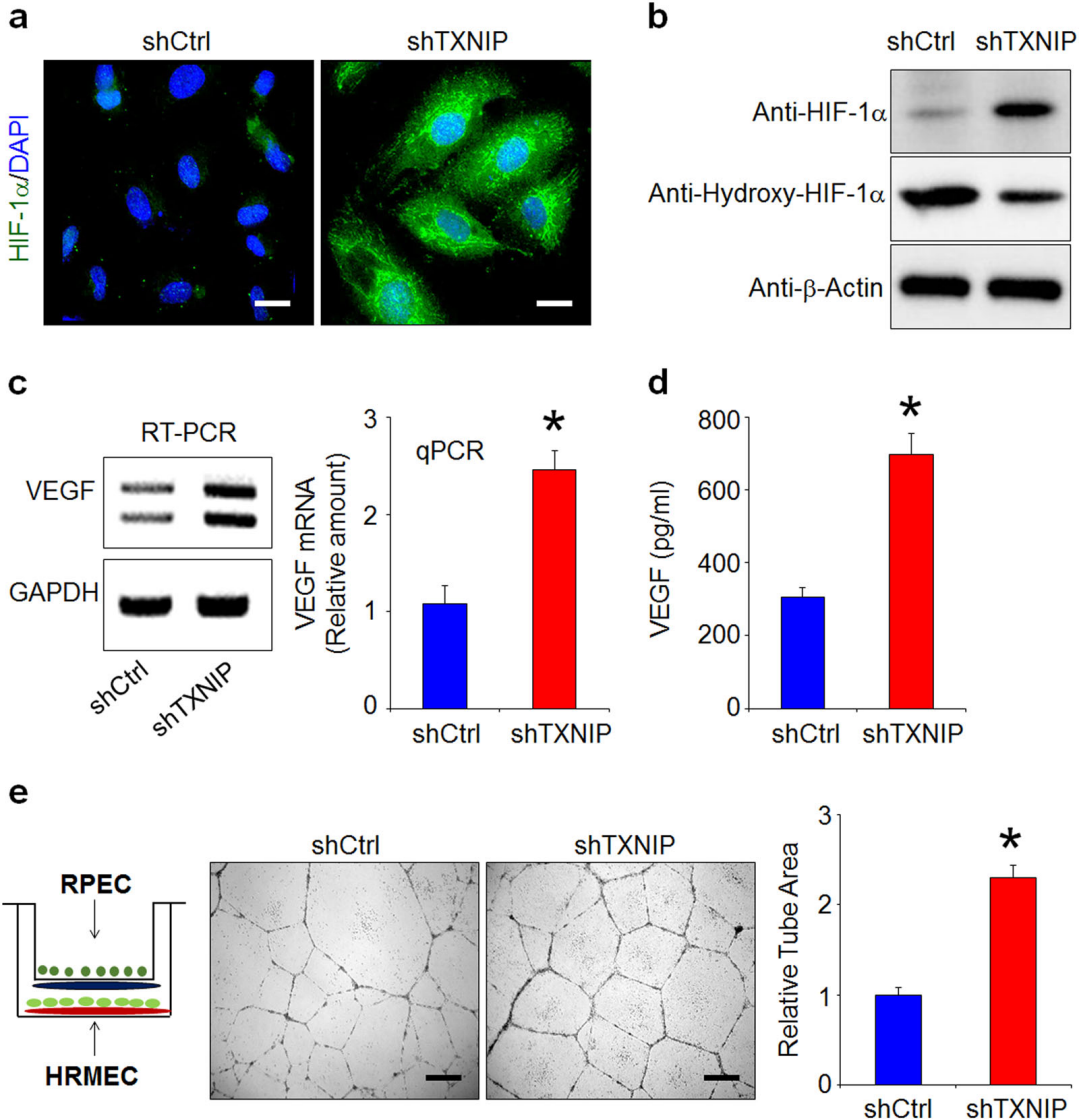
Loss of TXNIP increases HIF-1α and VEGF expression. **a** RPE cells were plated on coverslips coated with 10 μg/ml fibronectin. Immunostaining was performed on fixed cells with an anti-HIF-1α antibody. **b** RPE cells were lysed and blotted with the indicated antibodies. **c** The expression level of VEGF in RPE cells was analyzed by reverse transcription PCR (RT-PCR, left panel) and qPCR (right panel). **d** The secretion level of VEGF in RPE cells was determined by ELISA. Each experiment was performed in triplicate and repeated three times to assess the reproducibility of the results. **e** Representative images of capillary-like tube formation in HRMECs induced by coculture with RPE cells after 18 h. The relative wound and tube areas were calculated using ImageJ software. *p < 0.01 versus shCtrl. The error bars indicate the SEM. Scale bars, 30 μm (**a**) and 100 μm (**e**)

Oxidative stress increases HIF-1α expression and prevents its hydroxylation, which ultimately leads to the increased expression of VEGF, the major growth factor that triggers CNV in wet AMD^33,34^. To determine whether TXNIP can regulate HIF-1α expression in RPE cells, we performed immunostaining for HIF-1α. As shown in Fig. 5a, minimal HIF-1α expression was observed in the cytoplasm of control cells. However, the loss of TXNIP in ARPE-19 cells significantly increased HIF-1α expression in both the nucleus and cytoplasm. Consistent with this, western blot analysis showed that HIF-1α expression was increased, whereas the proline hydroxylation of HIF-1α was decreased in TXNIP-depleted ARPE-19 cells (Fig. 5b). Next, to determine whether the increased HIF-1α expression in TXNIP-depleted ARPE-19 cells has an effect on VEGF expression and secretion, we first evaluated the VEGF expression level. TXNIP-depleted cells displayed significantly increased VEGF expression compared with that displayed by control cells (Fig. 5c). Moreover, VEGF secretion was also increased in TXNIP-depleted ARPE-19 cells (Fig. 5d). Similarly, treatment with H_2_O_2_ in RPE cells also increased VEGF expression and secretion levels (Supplementary Fig. 4a, b). We also investigated whether the increase in VEGF secretion induces angiogenesis via paracrine activation. Tubule network formation by human retinal microvascular endothelial cells (HRMECs) cocultured with TXNIP knockdown cells was enhanced relative to that observed in control cells (Fig. 5e). These results indicate that the reduction in TXNIP expression levels in RPE cells by chronic or sustained oxidative stress might promote CNV through the induction of VEGF.

## Discussion

In this study, we showed that the downregulation of TXNIP causes RPE cell dysfunction via at least three pathways: (1) the induction of RPE cell autophagy through p53-mediated AMPK activation; (2) an increase in tight junction disruption and cell motility through Src kinase activation; and (3) the induction of CNV by HIF-1α-mediated VEGF secretion. Together, these pathways, which are regulated by TXNIP scaffold properties, lead to accelerated AMD pathogenesis. The results of the current study therefore indicate that TXNIP might serve as a therapeutic target for AMD by means of the maintenance of RPE functions.

Oxidative stress has been implicated in AMD pathogenesis, to which autophagy can contribute through the deregulation of cellular defense against such stress. However, aberrant autophagy has been frequently found to be associated with AMD; moreover, autophagy itself is considered a type of programmed cell death, supporting its involvement in cell death in AMD^35,36^. The tumor suppressor protein p53 acts as a key trigger for the induction of autophagy through AMPK activation^25^. Once cellular energy becomes depleted, AMPK is activated and then inhibits mTORC1 through the phosphorylation of TSC2 and raptor, thereby reducing ULK1 phosphorylation at Ser^757^. However, the activation of ULK1 at Ser^555^ and Thr^575^ by AMPK promotes autophagy^37^. Consistent with these phenomena, we observed that the activation of AMPK at Thr^172^ and ULK1 at Ser^555^ was increased in TXNIP-depleted ARPE-19 cells, whereas the phosphorylation of mTOR at Ser^2448^ and ULK1 at Ser^757^ was markedly decreased (Fig. 3d), suggesting that the depletion of TXNIP in ARPE-19 cells induced autophagy through p53-mediated AMPK activation. However, the method by which TXNIP regulates p53 activation remains unclear.

The activation of p53 occurs through multiple mechanisms, including increased protein concentration resulting from decreased p53 degradation, nuclear translocation, and posttranslational modifications such as phosphorylation and acetylation^38–40^. In particular, the phosphorylation of p53 at Ser^15^ blocks p53 binding with MDM2^27^. Accumulating evidence supports that p53 is inhibited during normal cell growth by MDM2 through either ubiquitin-dependent p53 degradation in the cytoplasm^26^ or the repression of p53 transcriptional activity in the nucleus^41^. In the present study, we also observed that the loss of TXNIP notably increased the phosphorylation of p53 at Ser^15^ and inhibited p53 binding with MDM2 (Fig. 3g, h). Under normal conditions, p53 is synthesized in the cytoplasm and transported between the cytoplasm and the nucleus in a cell cycle-dependent manner. It accumulates in the cytoplasm during G1 phase, localizes to the nucleus during the transition from G1 to S phase and is shuttled back to the cytoplasm shortly after the start of S phase^39^. However, our study showed that p53 was strongly expressed in the nucleus in TXNIP-depleted ARPE-19 cells. Moreover, p53-mediated cell growth suppression occurs via the transcriptional activity of the p53 target gene *CDKN1A*, which encodes p21^42^. Our study clearly showed that TXNIP loss significantly increased p21 expression levels, indicating that the sustained activation of p53 in the nucleus inhibits cell growth through p21 induction.

TXNIP constitutes a scaffold protein that serves as a multifunctional adaptor protein. Mutation in the chromosome region maintenance 1 (CRM1) binding domain of TXNIP suppresses HIF-1α nuclear export in cancer cells^43^. Poly (ADP-ribose) polymerase 1-mediated poly(ADP-ribosyl)ated p53 is unable to undergo nuclear export, as its interaction with CRM1 is impeded, which promotes the accumulation of p53 in the nucleus, where p53 exerts its transcriptional function^44^. Moreover, TXNIP plays important roles in the maintenance of hematopoietic cells. TXNIP interacts directly with p53 via cysteine residues and regulates p53 stability^45^. Thus, we suggest that the activation of p53 might be derived from the blockage of binding with MDM2 or the loss of the CRM1 binding domain of TXNIP, which causes p53 localization to the nucleus.

Tight junctions in RPE cells contribute to a restricted diffusion barrier between the retinal and choroidal perfusion. Oxidative stress has been reported to affect the disruption of RPE junctional proteins. The distribution of ZO-1 and occludin in tight junctions and cadherin in adherens junctions are disrupted by oxidative stress^2,46^. In the current experimental model, the depletion of TXNIP was associated with a defect in cell tight junctions and increased cell motility. This may be related to the RPE detachment observed during early AMD^47^. In our study, the downregulation of TXNIP by oxidative stress resulted in the disruption of ZO-1 along with the reorganization of perijunctional actin rings and an increase in actin stress fiber formation. However, the molecular mechanism by which oxidative stress mediates tight junction disruption in RPE cells remains unknown. In colorectal cancer cells, the oxidative stress-induced disruption of tight junctions is mediated by the activation of cSrc^48^. The TXNIP domain contains Src homology 3 binding domains and PPxY motifs. PPxY motifs in TXNIP interact with Src homology phosphatase-2 (SHP2), which directly regulates C-terminal Src kinase activation. In turn, the activation of SHP2 increases the phosphorylation of Src at Tyr^527^, which inhibits cSrc activity^49^. Herein, we propose that TXNIP-mediated Src kinase activation regulates RPE cell integrity. This is consistent with a report by Spindel et al. that showed that the downregulation of TXNIP in endothelial cells increases the phosphorylation of Src at Tyr^416^ through the regulation of SHP-2 plasma membrane localization, which results in increased actin stress fibers and the disruption of cell-to-cell junctions^50^.

The retina is the most metabolically active tissue in the human body and is highly sensitive to a reduction in oxygen tension. Therefore, any disturbances in oxygen delivery into the retina or local occlusive vascular diseases associated with inflammation in the eye ultimately leads to hypoxic conditions in the retina that may elicit the development of AMD^51–53^. Moreover, ROS and HIF-1α are directly involved in stimulating angiogenesis, both in tumors and in the retina^54,55^. ROS increase the gene expression of the nuclear transcription factor HIF-1α and prevent the hydroxylation of the HIF-1α protein, which is necessary for the elicited transcriptional activity of factors such as VEGF, a major stimulator of CNV. Consistent with this, VEGF is strongly expressed in surgically excised CNV membranes from human AMD eyes^56^. However, the mechanism by which oxidative stress mediates HIF-1α expression in RPE cells has not been clearly resolved. A previous study showed that the CRM1 region of TXNIP binds with HIF-1α and the ubiquitin ligase von Hippel-Lindau tumor suppressor (pVHL) and that the TXNIP-pVHL-HIF-1α complex is able to promote HIF-1α degradation^43^. Furthermore, the suppression of TXNIP by microRNA-224 leads to the nuclear translocation of HIF-1α^57^. In accordance with these results, our study showed that TXNIP is a negative regulator of HIF-1α expression, independent of its interaction with TRX and ROS. We therefore suggest that TXNIP can form a complex with HIF-1α, which leads to HIF-1α degradation, along with the inhibition of its nuclear translocation and ability to upregulate VEGF.

In conclusion, our results demonstrate that TXNIP plays a significant role in the regulation of p53-mediated autophagy induction and Src kinase-mediated tight junction disruption. Moreover, TXNIP regulates the activation of the transcriptional properties of HIF-1α, leading to increased VEGF secretion, which might promote CNV. Therefore, the dysfunction of RPE cells by a redox-dependent reduction in TXNIP is likely due to a reduction in the scaffold properties of TXNIP. Together, these findings support the potential role of TXNIP as a therapeutic target for AMD.

## Supplementary information

Supplementary Figs
