# Supplementary material for: Oxidative stress-mediated TXNIP loss causes RPE dysfunction
Source: Exp Mol Med. 2019 Oct 15;51(10):121. doi: 10.1038/s12276-019-0327-y (PMC6802648; doi:10.1038/s12276-019-0327-y)
Supplement: Supplementary file 1 — Supplementary Figs [file 12276_2019_327_MOESM1_ESM.docx]

**Supplementary data to:**

**Oxidative stress-mediated TXNIP loss causes RPE dysfunction**

**Running title: TXNIP regulates RPE homeostasis**

Min Ji Cho^1,2^ *, Sung-Jin Yoon^3,^*, Wooil Kim^1,2^, Jongjin Park^1^, Jangwook Lee^1^, Jong-Gil Park^1^, Young-Lai Cho^4^, Jeong Hun Kim^5^, Hyejin Jang^2,3^, Young-Jun Park^2,3^, Sang-Hyun Lee^1, §^,

and Jeong-Ki Min^1,2, §^

**Affiliations**

^1^ Biotherapeutics Translational Research Center, Korea Research Institute of Bioscience and Biotechnology (KRIBB), 125 Gwahak-ro, Yuseong-gu, Daejeon 34141, Republic of Korea

^2^ Department of Biomolecular Science, KRIBB School of Bioscience, Korea University of Science and Technology (UST), 217 Gajeong-ro, Yuseong-gu, Daejeon 34141, Republic of Korea

^3^ Environmental Disease Research Center, Korea Research Institute of Bioscience and Biotechnology (KRIBB), 125 Gwahak-ro, Yuseong-gu, Daejeon 34141, Republic of Korea

^4^ Metabolic Regulation Research Center, Korea Research Institute of Bioscience and Biotechnology (KRIBB), 125 Gwahak-ro, Yuseong-gu, Daejeon 34141, Republic of Korea

^5^ Fight against Angiogenesis-Related Blindness (FARB) Laboratory, Clinical Research Institute, Seoul National University Hospital, 101 Daehak-ro, jongno-gu, Seoul 03080, Republic of Korea

* These authors contributed equally to this work.


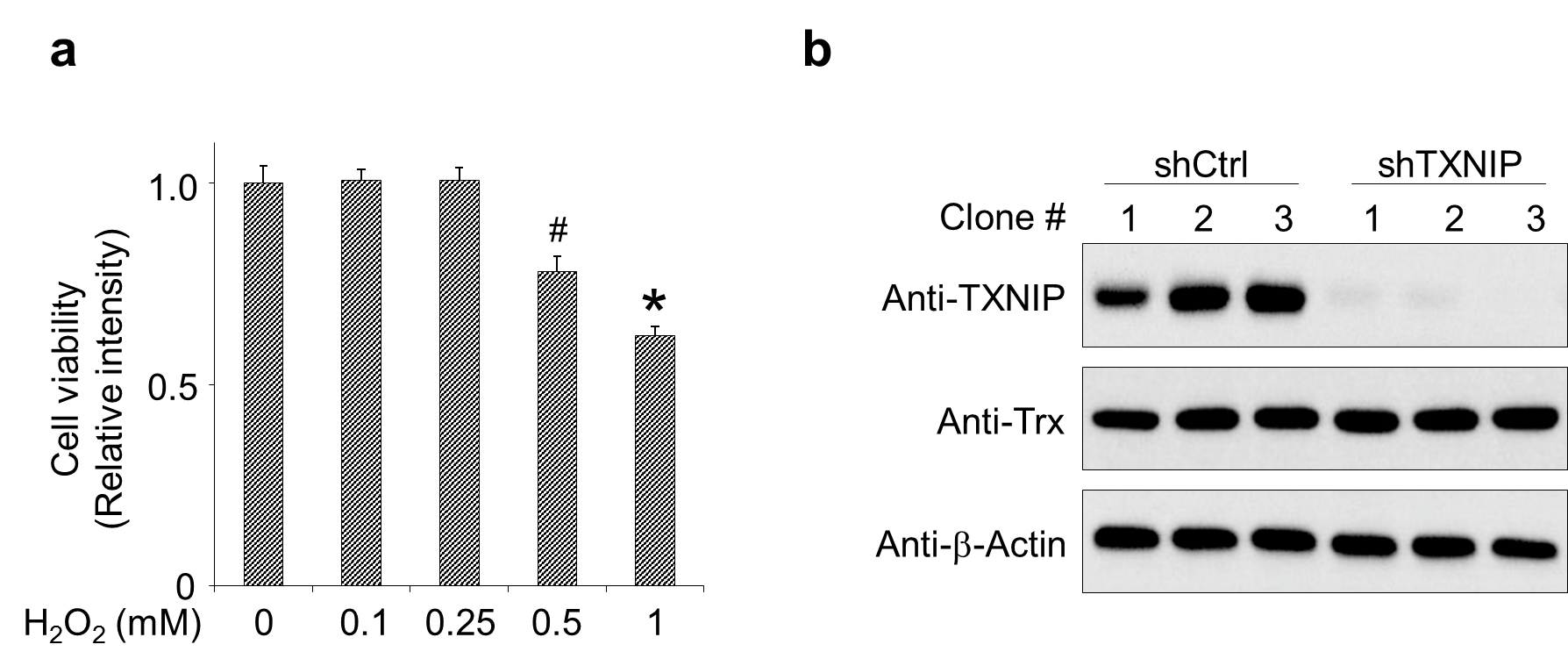


**Supplementary Fig. 1. a** ARPE-19 cells were treated with indicated concentration of H_2_O_2_ for 48 hr. MTT assay of the ARPE-19 cells viability after treatment with H_2_O_2_, compared with that of the without H_2_O_2_ treatment. The results shown are representative of three times independent experiments. **b** TXNIP shRNA lentivirus was infected in ARPE-19 cells. **p* < 0.01; ^#^*p* < 0.05 versus without H_2_O_2_. Error bars indicate ± SEM.


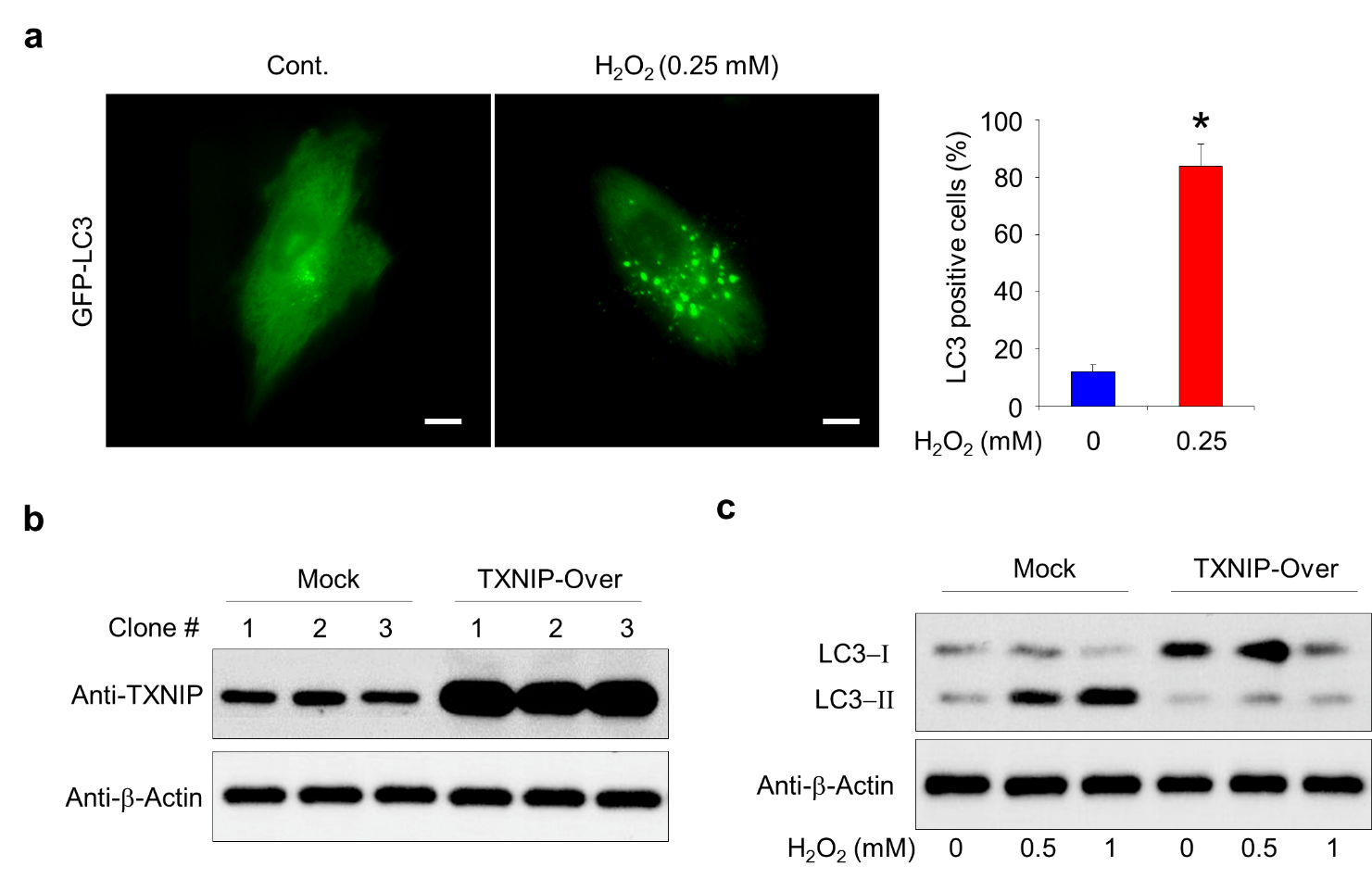


**Supplementary Fig. 2. a** ARPE-19 cells were transfected with GFP-LC3. After transfection, cells were plated on coverslips coated with 10 μg/ml fibronectin and treated with 0.25 mM H_2_O_2_ for 4 hr. The positive cells of LC3 puncta were counts from the images (%, LC3 positive cells / total cells). **b** TXNIP over-expression lentivirus was infected in ARPE-19 cells. **c** RPE cells were treated with indicated concentration of H_2_O_2_ for 4 hr. Cells were lysed and subjected to western blot by indicated antibodies. **p* < 0.01 versus without H_2_O_2_. Error bars indicate ± SEM.


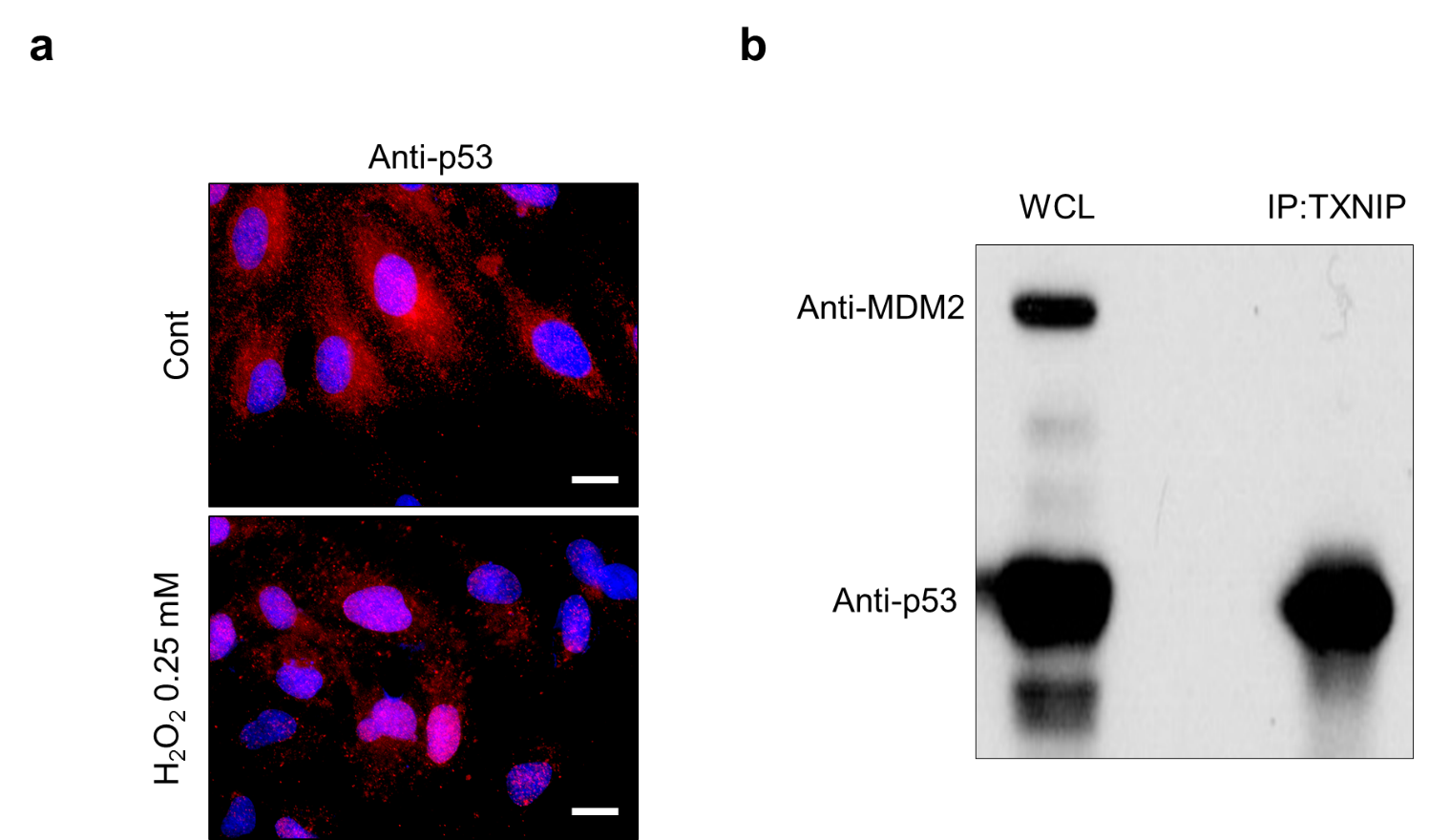


**Supplementary Fig. 3. a** ARPE-19 cells were treated with 0.25 mM H_2_O_2_ for 4 hr. Immunostaining was performed on fixed cells with anti-p59 antibody. **b** Cell lysates were immunoprecipitated with an anti-TXNIP antibody, blotted, and probed with an anti-MDM2 and anti-p53 antibodies.


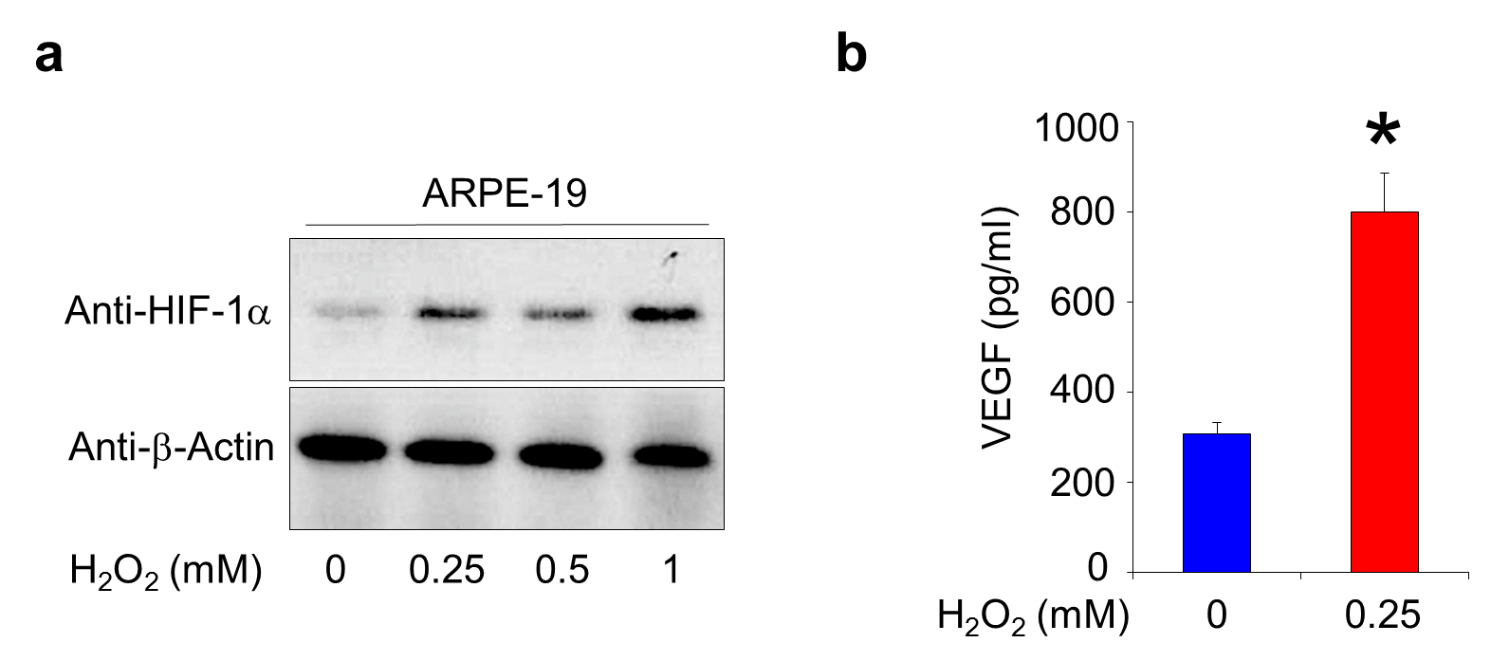


**Supplementary Fig. 4. a** ARPE-19 cells were treated with indicated concentration of H_2_O_2_ for 8 hr. Cells were lysed and subjected to western blot by indicated antibodies. **b** The secretion levels of VEGF in RPE cells were determined by ELISA. RPE cells were stimulated with 0.25 mM H_2_O_2_ for 4 hr. After stimulation for 4 hr, the supernatant collected and then performed ELISA assay. Each experiment was performed in triplicate and repeated three times to assess for reproducibility of the results. **p* < 0.01 versus without H_2_O_2_. Error bars indicate ± SEM.
